# The Small Secreted Protein FoSsp1 Elicits Plant Defenses and Negatively Regulates Pathogenesis in *Fusarium oxysporum* f. sp. *cubense* (Foc4)

**DOI:** 10.3389/fpls.2022.873451

**Published:** 2022-05-10

**Authors:** Yuhua Wang, Xinchun Zhang, Tian Wang, Siyu Zhou, Xiaofei Liang, Changping Xie, Zhensheng Kang, Daipeng Chen, Li Zheng

**Affiliations:** ^1^Key Laboratory of Green Prevention and Control of Tropical Plant Disease and Pests, Ministry of Education and School of Plant Protection, Hainan University, Haikou, China; ^2^Environment and Plant Protection Institute, Chinese Academy of Tropical Agricultural Sciences, Haikou, China; ^3^State Key Laboratory of Crop Stress Biology for Arid Areas and College of Plant Protection, Northwest A&F University, Yangling, China

**Keywords:** *Fusarium oxysporum*, secreted protein, conidiation, pathogenesis, elicitor

## Abstract

Fusarium wilt of banana (*Musa* spp.), a typical vascular wilt disease caused by the soil-borne fungus, *Fusarium oxysporum* f. sp. *cubense* race 4 (Foc4), seriously threatens banana production worldwide. Pathogens, including vascular wilt fungi, secrete small cysteine-rich proteins during colonization. Some of these proteins are required for pathogenicity. In this study, 106 small secretory proteins that contain a classic N-terminal signal peptide were identified using bioinformatic methods in Foc4. Among them, 11 proteins were selected to show transient expressions in tobacco. Interestingly, transient expression of FoSsp1 in tobacco, an uncharacterized protein (of 145 aa), induced necrotic cell death reactive oxygen burst, and callous deposition. Furthermore, the expression of *FoSSP1* in Foc4 wild type (WT) was up-regulated during the stage of banana roots colonization. A split-marker approach was used to knock out *FoSSP1* in the Foc4 WT strain. Compared with the WT, the deletion mutant *Fossp1* was normal in growth rate but increased in conidiation and virulence. RT-qPCR analysis showed that the expression of four conidiation regulator genes in the *Fossp1* deletion mutant was significantly decreased compared to the WT strain. In addition, the expression of four pathogenesis-related genes of bananas infected with *Fossp1* deletion mutant was down-regulated in comparison with that of the WT. In summary, these results suggested that *FoSSP1* is a putative elicitor that negatively regulates conidiation and pathogenicity in Foc4.

## Introduction

Fusarium wilt of bananas is known as “Panama disease”, damaging the banana production around the world (Hwang and Ko, 2004). The causing agent *Fusarium oxysporum* f. sp. *cubense* (Foc) can be further divided into three races (Foc1, 2, and 4) based on pathogenicity differences to banana cultivars. Among them, Foc4 is the most virulent one which can infect all Cavendish banana cultivars in addition to all the cultivars that are susceptible to Foc1 and Foc2 (Ploetz et al., 2015). Foc4 can be further divided into tropical race 4 (TR4) and subtropical race 4 (STR4), of which TR4 is more virulent (Butler, 2013). TR4 was reclassified into the new species *F. odoratissimum*, based on the genetic diversity of Foc isolates in the Indonesian center of origin (Maryani et al., 2019). As a soil-borne fungus, Foc usually infects the root first. Conidia or hyphae move from soil to the surface of the root and rhizome, and then hyphae penetrate the root xylem and spread to the pseudostem xylem and even the outer leaf sheaths. At last, the infected plant shows symptoms (Dita et al., 2018). Pathogen spreading in the vascular bundle of bananas blocks nutrients and water transportation, resulting in leaves yellowing and plant death (Michielse and Rep, 2009).

In nature, there is a constant arm race between plants and pathogens. These pathogens use numerous virulence factors for pathogenicity or fitness in the plant (Chisholm et al., 2006). Therefore, plants need to mount effective defense mechanisms to protect themselves, including two innate immune systems (Boller and Felix, 2009; Dou and Zhou, 2012). When pathogens attack host cells, the pattern-recognition receptors (PRRs) of cell membranes are the first to detect the pathogen by recognizing pathogen-associated molecular patterns (PAMPs) of pathogens (Bigeard et al., 2015). This nonspecific immunity called Pattern-Triggered Immunity (PTI) can halt further colonization (Jones and Dangl, 2006). This is the first line of defense in plants against pathogens. In response, the pathogen secretes effectors which can interfere with PTI and result in effector-triggered susceptibility (ETS) (Bu et al., 2014). At the same time, the resistance protein of the plants detects the effectors to trigger the specific immunity of the plant, which is the second line of defense (known as ETI, Effector-Triggered Immunity) (Tsuda and Katagiri, 2010).

Currently, it has been reported that small secreted proteins play an important role in the interactions between plants and the pathogen. There are more and more studies on small secreted proteins, which share the same characteristics: generally, <300 amino acids, lack transmembrane domain, contain an N-terminal signal peptide (SP), and rich-in cysteine (Niu et al., 2021). Fungal small secreted proteins involved in the interaction between pathogens and plants have been referred to as effectors or elicitors. Recently, those effectors or elicitors have been identified in many fungal pathogens. PstSCR1 as an elicitor in wheat rust pathogen, *Puccinia striiformis* f. sp. *tritici*, can activate the surface-mediated immunity in *Nicotiana benthamiana* which contributes to the non-host resistance to oomycete pathogens (Dagvadorj et al., 2017). As an effector, CfEC92 inhibits plant immunity and participates in the early infection stage of *Colletotrichum fructicola* to glomeruli (Shang et al., 2020). VmE02 has been reported as a novel cross-kingdom PAMP widely spread in fungi and oomycetes, which exhibited cell-death-inducing activity in *N. benthamiana*. However, deletion of VmE02 in *Valsa mali* has no effect on pathogenicity but significantly reduces conidia production (Nie et al., 2019b).

Here, in this study, 106 small secretory proteins that contain a classic N-terminal signal peptide, were identified by bioinformatic methods in Foc4. Among them, 11 proteins were selected for transient expression in tobacco. Interestingly, transient expression of FoSsp1, an uncharacterized protein (of 145 aa), induced necrotic cell death reactive oxygen burst, and callous deposition in *N. benthamiana*. Furthermore, the expression of *FoSSP1* was up-regulated during the stage of banana roots colonization. A split-marker approach was used to knock out *FoSSP1* in the Foc4 wild type (WT) strain. Compared with the wild type, the deletion mutant *Fossp1* was normal in growth rate but increased in conidiation and virulence. RT-qPCR analysis showed that the expression of four conidiation regulator genes in the *Fossp1* deletion mutant was significantly decreased compared to the WT strain. In addition, the expression of four pathogenesis-related (PR) genes of bananas infected with *Fossp1* deletion mutant was down-regulated in comparison with that of the wild type. In summary, these results suggested that *FoSSP1* is a putative elicitor that negatively regulates conidiation and pathogenicity in Foc4.

## Materials and Methods

### Strains and Plant Growth Conditions

The WT strain of *F. oxysporum* f. sp. *cubense* used in the experiment was Foc4. The mycelium was maintained on potato dextrose Agar (PDA) medium and the conidial suspension was stored in 24% glycerol at −80 °C. The knockout and complement transformants were cultured on a medium containing 50 mg/mL hygromycin B or G418, respectively. *E. coli* DH5a, BL21 and *A. tumefaciens* GV3101, AGL1 were grown on a lysogeny broth (LB) medium containing corresponding antibiotics and were stored in 24% glycerol at −80°C. *N. benthamiana* was grown in a greenhouse with 8/16 (light/dark) per day at 24°C. *Musa* AAA, as warm-loving wet crops, was cultivated in a warm shed with a temperature of 30°C.

### Plasmid Construction

To investigate whether FoSsp1 can induce hypersensitivity response in *N. benthamiana*, Phusion Plus DNA polymerase (Thermo Fisher Scientific) was used to amplify the coding region of *FoSSP1* and the coding region without signal peptide *FoSSP*1^Δ*SP*^ from the cDNA of Foc4 and cloned into the plant expression vector pBin-eGFP, respectively. The same sequence was amplified by PCR and cloned into a pSUC2 vector using *Eco*RI and *Xho*I restriction sites by ClonExpress^®^ II One Step Cloning Kit (Vazyme Biotech) for verifying the secretion activity of signal peptide (SP) (Oh et al., 2009). The encoding region and promoter sequence of *FoSSP1* were amplified from WT genomic DNA and cloned into the pFL2 vector to obtain the complementary vector pFL2-HB (Chen et al., 2014).

### Total RNA Extraction and Quantitative Reverse Transcription PCR

To verify the expression of *FoSSP1* during the process of the plant infection, roots of banana seedlings were inoculated with conidial suspension with a concentration of 10^8^/mL. Total RNAs were extracted from the inoculated roots (12, 24, 36, 48, 60, and 72 hpi). The relative expression of *FoSSP1* in conidia was considered as the 0 hpi control. The extraction method refers to Eastep® Super Total RNA Extraction Kit (Promega), following the manufacturer's instructions. The cDNA was synthesized with the PrimeScript 1st strand cDNA Synthesis kit (Takara), according to the manufacturer's instructions.

A pair of specific (SSP1-RT-F/R) primers of the gene was used for qRT-PCR analysis with 100 ng cDNA as a template (Supplementary Table S1). The qRT-PCR was performed using the SuperReal PreMix Plus kit (Tiangen Biotech), following the manufacturer's instructions. The actin gene was used as an endogenous reference to normalize the expression of *FoSSP1* in different periods and the relative expression levels of genes were calculated using the 2^−Δ*ΔCt*^ method (Livak and Schmittgen, 2001). Select one of the sets of data and arbitrarily set its relative expression to 1. The qRT-PCR conditions were as follows: an initial 95°C denaturation step for 3 min, followed by denaturation for 15 s at 95°C, annealing for 15 s at 57°C, and extension for 20 s at 72°C for 35 cycles (Wu et al., 2010). For the relative expression of PR genes in infected bananas, the actin gene (Degradi et al., 2021) of bananas was used as an endogenous reference to normalize the expression. One-way ANOVA was used to compare the significance of the difference between the two groups at the same time point.

### Signal Peptide Secretion Activity Verification

The recombinant vectors pSUC2-*FoSSP1* and pSUC2-*FoSSP*1^Δ*SP*^ were constructed and transferred into the YTK12 strain (Gu et al., 2011). The CMD-W medium (0.08% tryptophan dropout supplement, 0.65% yeast nitrogen base without amino acids, 2% sucrose, 0.1% glucose, and 2% agar) was used to screen the positive colonies from all the transformants. The successful transformant was cultured on a YPRAA medium to verify its secretion activity. To further test the secretion activity of the signal peptide, the reduction of 2, 3, 5-triphenyltetrazolium chloride (TTC) to insoluble red-colored 1, 3, 5-triphenylformazan (TPF) was monitored.

### Culture Conditions and Fungal Transformation

Gene replacement was carried out according to homologous recombination (Yang et al., 2018b). The upstream and downstream flanking sequences of *FoSSP1* were cloned with primer pairs of UF/UR and DF/DR (containing the Hyg tag) [(Supplementary Table S1) Polynucleotide chain reaction primers used in this study], using the DNA of Foc4 as a template. The upstream and downstream of hygromycin resistant gene fragment was amplified with PCR primer pair of HYG-F/R and pEX2 vector as DNA template. Fusion fragment for transformation was generated by overlap PCR as described (Goswami, 2012). Purified fragments for protoplast transformation using Gel Extraction Kit (Omega) according to the manufacturer's instructions.

The hyphae were digested in enzymatic solution (25 g/L Driselase, 5 g/L Lysing Enzymes in 1.2 M KCl) and protoplasts were released until the concentration is 10^7^ protoplasts/ml (Yun et al., 2014). The DNA fragments or plasmids for transformation were mixed with protoplasts and incubated on ice for 20 min. Then, 1 ml PTC solution (0.6 M KCl, 50 mM Tris-HCl pH 8.0, 50 mM CaCl_2_, and 40% PEG 4000) was added to the mixture and incubated at room temperature for 30 min. The mixture was transferred to a TB3 medium for further culture for 20 h. The transformed mixture was inoculated on a 15ml PDA medium containing 50 μg/ml hygromycin B or G418, then covered with PDA agar modified with 75 μg/ml hygromycin B or G418. Transformants harboring hygromycin B or G418 resistance were identified by PCR.

### Transient Expression Assay in *N. benthamiana*

The recombinant vectors pBin-*FoSSP1* and pBin-*FoSSP*1^Δ*SP*^ were transferred into *Agrobacterium tumefaciens* GV3101 by electroporation and cultured in LB containing 20 mg/ml rifampicin and 50 mg/ml kanamycin for 48 h. Bacteria were harvested and resuspended with injection buffer (1% MgCl_2_·6H_2_O, 1% MES, 150 μM acetosyringone) to the OD_600_ of 1.0. The suspension was incubated at room temperature for 2–3 h to release the plasmids in the injection buffer. A plant of *N. benthamiana* at the stage of four leaves was selected and infiltrated the buffer from the back of the leaves (Wei et al., 2020). The empty pBin-eGFP vector was used as a negative control, and the vector containing BAX was used as a positive control. Leaves cell death symptoms were photographed both under natural light and under ultraviolet (wavelength 365 nm) at 6 days post-inoculation. Each assay was repeated three times.

### Protein Extraction and Western Blot

Leaves were thoroughly ground in liquid nitrogen. The process of protein separation refers to the previous (Yang et al., 2012). The 20 μl total protein was separated with the 12.5% sodium dodecyl sulfate polyacrylamide gel electrophoresis (Shenggong Biotech) and transferred to the polyvinylidene difluoride (PVDF) membrane. Rabbit anti-GFP pAb (ABclonal) and rabbit anti-HA pAb (Shenggong Biotech) were used as primary antibodies and HRP Goat anti-rabbit IgG (ABclonal) as secondary antibodies.

### Phenotypic Characterization of the Foc Mutants

To explore the growth rate, *Fossp1*, WT, and *Fossp1*-C were inoculated on the PDA plates, respectively, and cultured at 28°C for 6 days. The size of fungus mycelium was measured by crossing every day to calculate the mycelium growth rate. WT, *Fossp1*, and *Fossp1*-C were cultured in a PDB medium at 28°C. Up to the fourth day, the conidia numbers were counted daily using a hemocytometer.

WT, *Fossp1*, and *Fossp1*-C were cultured in a PDB medium for 4 days, and the spores were collected at least 10^8^ conidia/ml. The roots of banana seedlings were cleaned and root wounds were made with an art knife, then immersed in the prepared spore solution for 30 min. The seedlings were transplanted into flowerpots. And 50 mL conidial suspension was poured into each pot.

To evaluate the disease index, the corm of banana seedlings at 20 dpi was cut to measure the lesion area. The lesion area was used for disease grade, disease grade for each plant ranging from 0 to 4 (0, no lesion in corm; 1, 1–10% lesion area in corm; 2, 11–30% lesion area in corm; 3, 31–50% lesion area in corm; 4, more than 50% lesion area in corm). The disease index of infected banana seedlings was calculated as follows formula: Disease index (%) = [∑ (grade × number of plants in that grade)/ (4 × total number of assessed plants)] × 100% (Zhang et al., 2019).

### Subcellular Localization in Tobacco Leaves

*Agrobacterium* containing recombinant vectors and pBin-*FoSSP*1^Δ*SP*^ was re-suspended in buffer solution at OD_600_ of 0.001. The syringe was used to infiltrate *Agrobacterium* solutions into the tobacco leaves. The leaves were photographed and observed under a laser confocal microscope 2 days post infiltration. DAPI staining was conducted as previously described (Tarnowski et al., 1991). The empty vector was set as the negative control.

### ROS Staining and Callose Deposition Detection

The accumulation of H_2_O_2_ was detected by staining tobacco leaves with 3, 3'-diamino benzidine (DAB). The leaves were immersed in *A. tumefaciens* for 3 days and stained in DAB for 12 h in the dark. Then the leaves were destained in 95% ethanol until translucent and photographed (Dong and Chen, 2013).

For callose staining, tobacco leaves were boiled and decolorized in 95% ethanol, and then immersed in 1% aniline blue solution in the dark for at least 2 h. The deposition of callose was detected under the microscope.

## Result

### FoSsp1 Is Conserved in Filamentous Fungi

**Figure 1:**
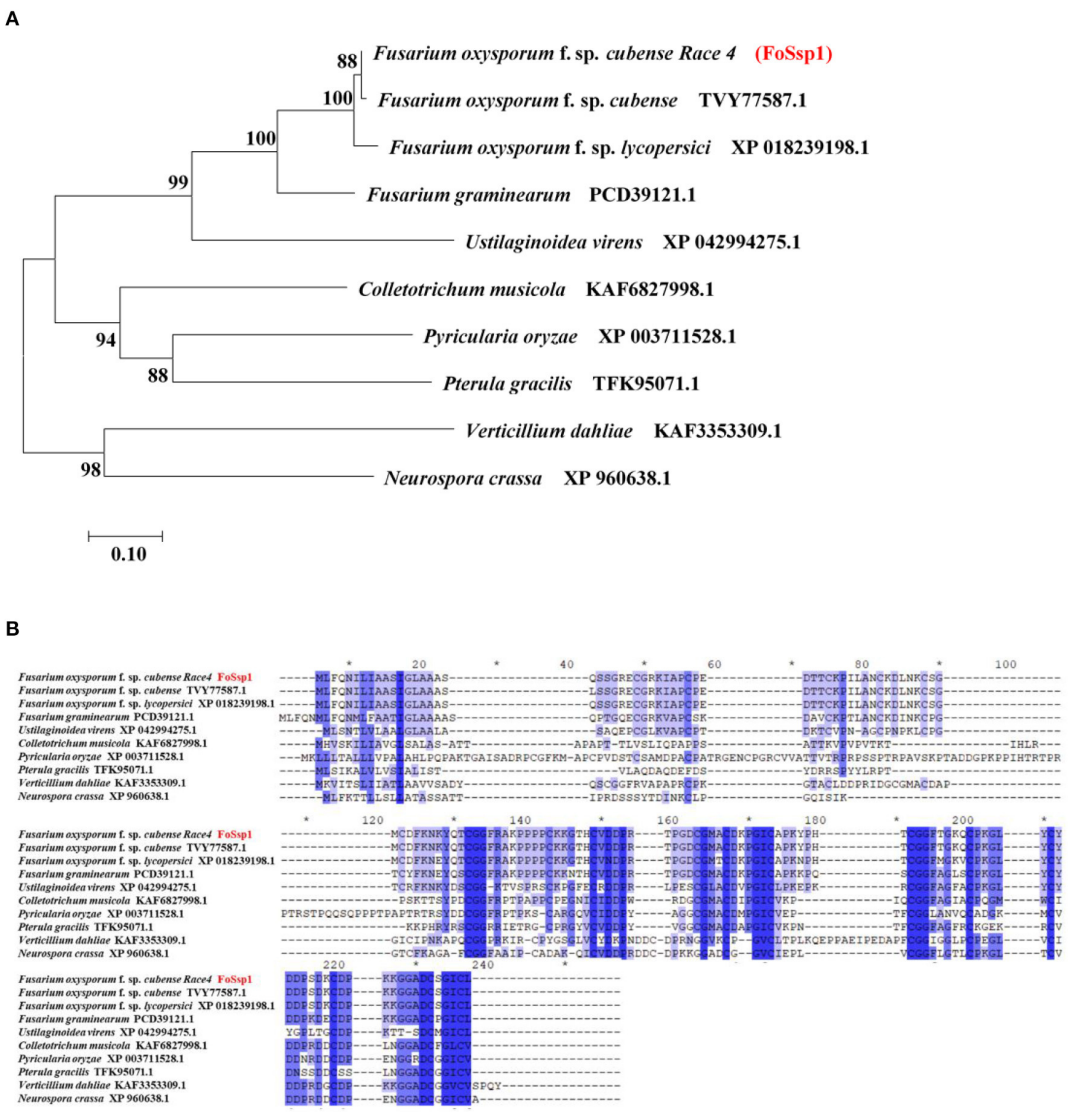
Homology analysis of Ssp1 protein among filamentous fungi. **(A)** The unrooted tree of full-length protein was generated with the MEGA7 program by the neighbor-joining method. Numbers on major branches indicate percentage boot strap confident scores (1,000 replications). The genetic distance corresponding to the scale is 0.10. **(B)** Comparative analysis of protein sequences of FoSsp1 and its homologous proteins. The protein sequences were compared and edited with GeneDoc, and the different shades of blue reflected the level of amino acid identity at each location. Identical and conserved catalytic residues are marked with an asterisk (^*^).

A small secreted protein of plant pathogens is characterized by an N-terminal signal peptide, no transmembrane domain, rich-in cysteine, and no more than 300-amino acids (Zhang et al., 2020). Here, SignalP (Nielsen, 2017) (https://services.healthtech.dtu.dk/service.php?SignalP-5.0), TMHMM (https://services.healthtech.dtu.dk/service.php?TMHMM-2.0) and Big-PI Predictor (https://mendel.imp.ac.at/gpi/fungi_server.html) were used to predict the small secreted proteins of *F. oxysporum* f. sp. *Cubense* Race 4 (Foc4). More than 1% of cysteine is considered rich in cysteine. In total, 106 putative secreted proteins were obtained that met the above characteristics. Among them, 11 proteins were selected randomly for transient expression in tobacco (Supplementary Table S2). Small secreted proteins screened in this study). The encoding genes were constructed into a recombinant pGR107 vector and transiently expressed in *N. benthamiana* leaves by *A. tumefaciens*-mediated transformation. The results showed that FOIG_06406 (Gene ID: 42031581) which encodes a 145-amino acids protein can induce necrotic cell death in *N. benthamiana* (**Figure 3A**). We named it *FoSSP1* (Small Secreted Protein) in this study. A BLASTp search against the NCBI Nr database was conducted using the FoSsp1 protein sequence (NCBI Reference Sequence: XP_031064189.1) as a query, results indicated that FoSsp1 homologs are conserved in the *Fusarium* genus and many other higher Ascomycetes. Interestingly, FoSsp1 homologs were also found in *Pterula gracilis*, which belongs to Basidiomycetes. These results indicate that FoSsp1 was conserved in fungi (Figure 1).

### The Expression of *FoSSP1* Was Up-Regulated in Foc4 Infected Banana Roots

**Figure 2:**
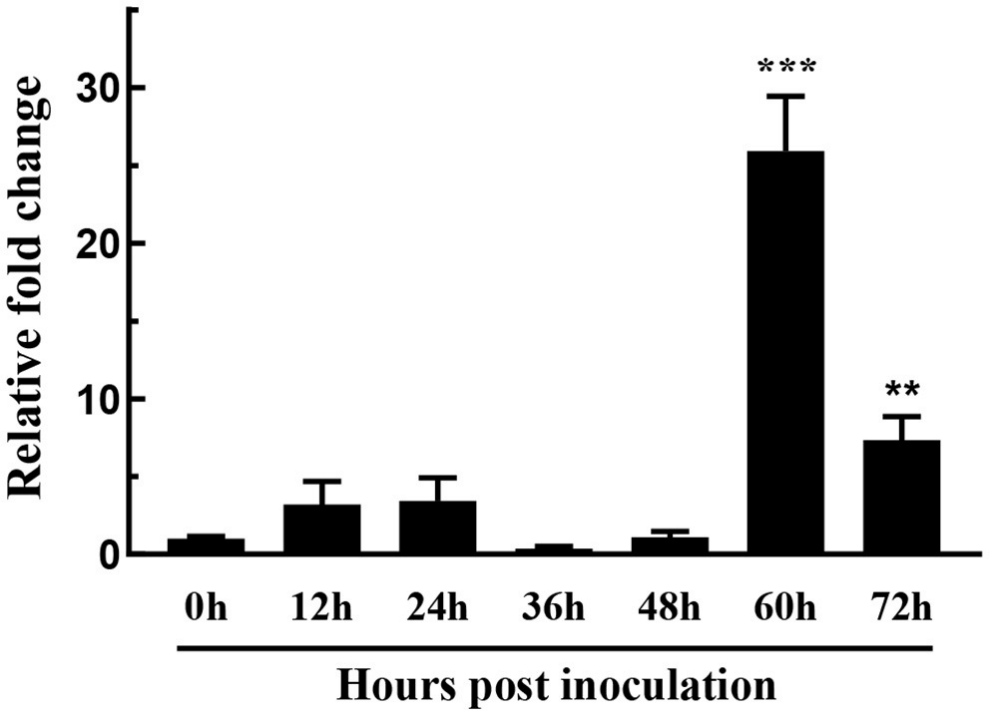
The expression level of *FoSSP1* in Foc4 WT during different times after being inoculated in banana roots (0, 12, 24, 36, 48, 60, and 72 hpi). The relative expression of *FoSSP1* in conidia (0 h control) was arbitrarily set to 1. The results are representative of three independent experiments. Error bars represent standard error. Asterisks indicate significant differences in one-way analysis of variance (*p* < 0.01).

Fungal secreted proteins usually participate in pathogen infection. To explore the relative expression of *FoSSP1*, qRT-PCR was conducted in banana roots infected with Foc4. Compared with the expression of *FoSSP1* in conidia (0 hpi, hour post-inoculation), the expression of *FoSSP1* was significantly up-regulated at 60 and 72 hpi (hours post-inoculation). There was no significant difference between the expression levels in other periods and the gene expression levels in spores (Figure 2). This result indicates that *FoSSP1* is induced in plant infection, implying its function in the plant-pathogen interaction.

### FoSsp1 Can Induce Cell Death of *N. benthamiana*

**Figure 3:**
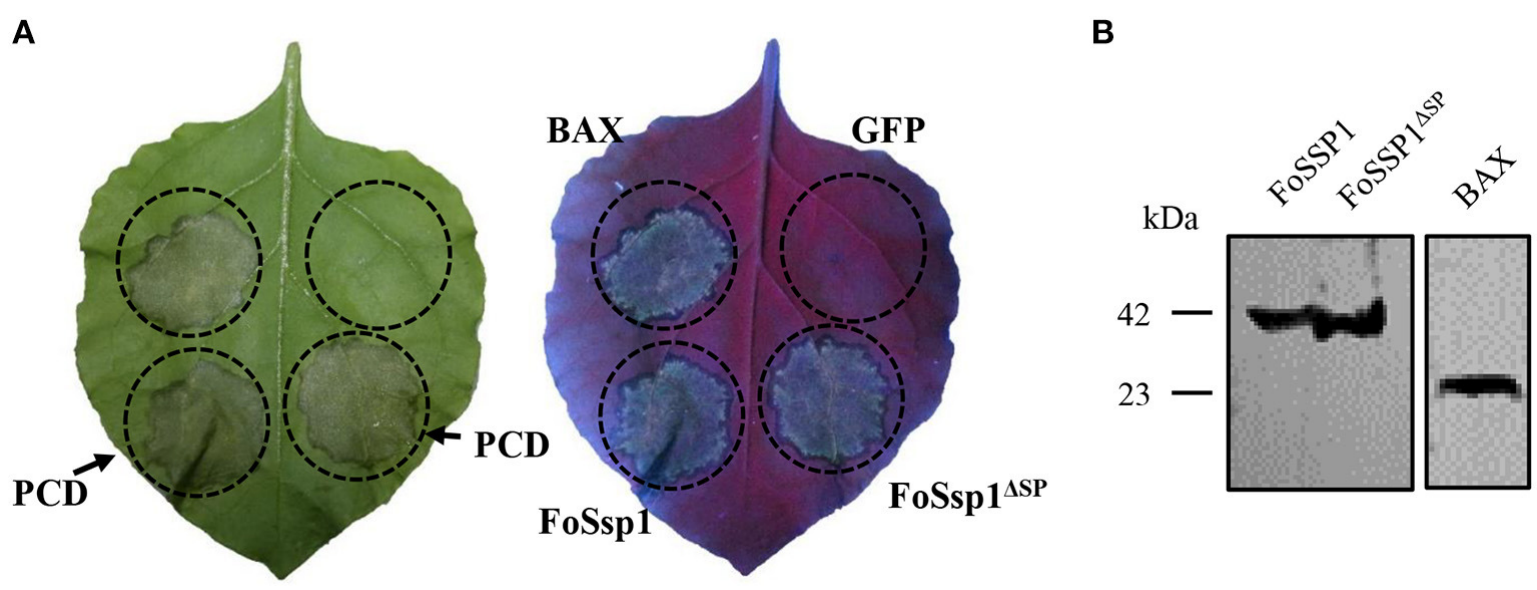
FoSsp1 was transiently expressed in the *N. benthamiana* by *A. tumefaciens*-mediated transformation. **(A)** FoSsp1 induced cell death in tobacco leaves. The recombinant vectors of pBin-*FoSSP1* and pBin-*FoSSP*1^Δ*SP*^ were infiltrated into leaves by *A. tumefaciens*-mediated transformation. Empty vector pBin-eGFP (EV) was used as negative control and Bax as positive control. After 5 days of infiltration, the hypersensitive response was indicated by ultraviolet (UV) light. The experiment was repeated at least four times. **(B)** Western blot analysis was used to detect the protein expression.

To dissect the effect of FoSsp1 on plant defense, transient expression of FoSsp1 in *N. benthamiana* was conducted. The cell death triggering protein Bax (Gene ID: 581) (Lacomme and Santa Cruz, 1999), was set as a positive control. Given that signal peptide is often thought to be cleaved to form mature proteins (Owji et al., 2018), recombinant vectors of pBin-eGFP-*FoSSP1* and pBin-eGFP-*FoSSP*1^Δ*SP*^ were constructed respectively. Results indicate that Bax, pBin-eGFP-*FoSSP1*, and pBin-eGFP-*FoSSP*1^Δ*SP*^ induced cell death in leaves of *N. benthamiana* after 5 days post agroinfiltration, whereas the EV (empty vector) did not (Figure 3A). Proper protein expression in *N. benthamiana* leaves was further confirmed by Western Blot analysis (Figure 3B). The experiment was repeated three times with the same results. The above results showed that FoSsp1 could induce cell death in *N. benthamiana*.

### *FoSSP1* Deletion Mutant Was Normal in Growth Rate but Increased in Conidiation

Split-marker approach was used to generate the deletion mutant of *FoSSP1* in Foc4. The nucleotide sequence of a segment containing the *FoSSP1* gene (NW_022158695.1: 156,986-157,890 nt) was replaced by a hygromycin-resistance gene in Foc4. The resulting transformants harboring the hygromycin B resistance were further confirmed by PCR. In total, five strains of *Fossp1* deletion mutant were generated and FT1 was chosen for further experiments [(Supplementary Figure S1) PCR Test of *Fossp1* deletion mutants]. To generate the complement strain of the *Fossp1* mutant, the promoter and the encoding region of *FoSSP1* (NW_022158695.1: 157,201-157,734 nt) were constructed into the pFl2 plasmid and transformed into the *Fossp1* deletion mutant by PEG-mediated protoplast transformation. The resulting transformants harboring neomycin (G418) resistance were screened by PCR. In total, six complement strains were generated.

**Figure 4:**
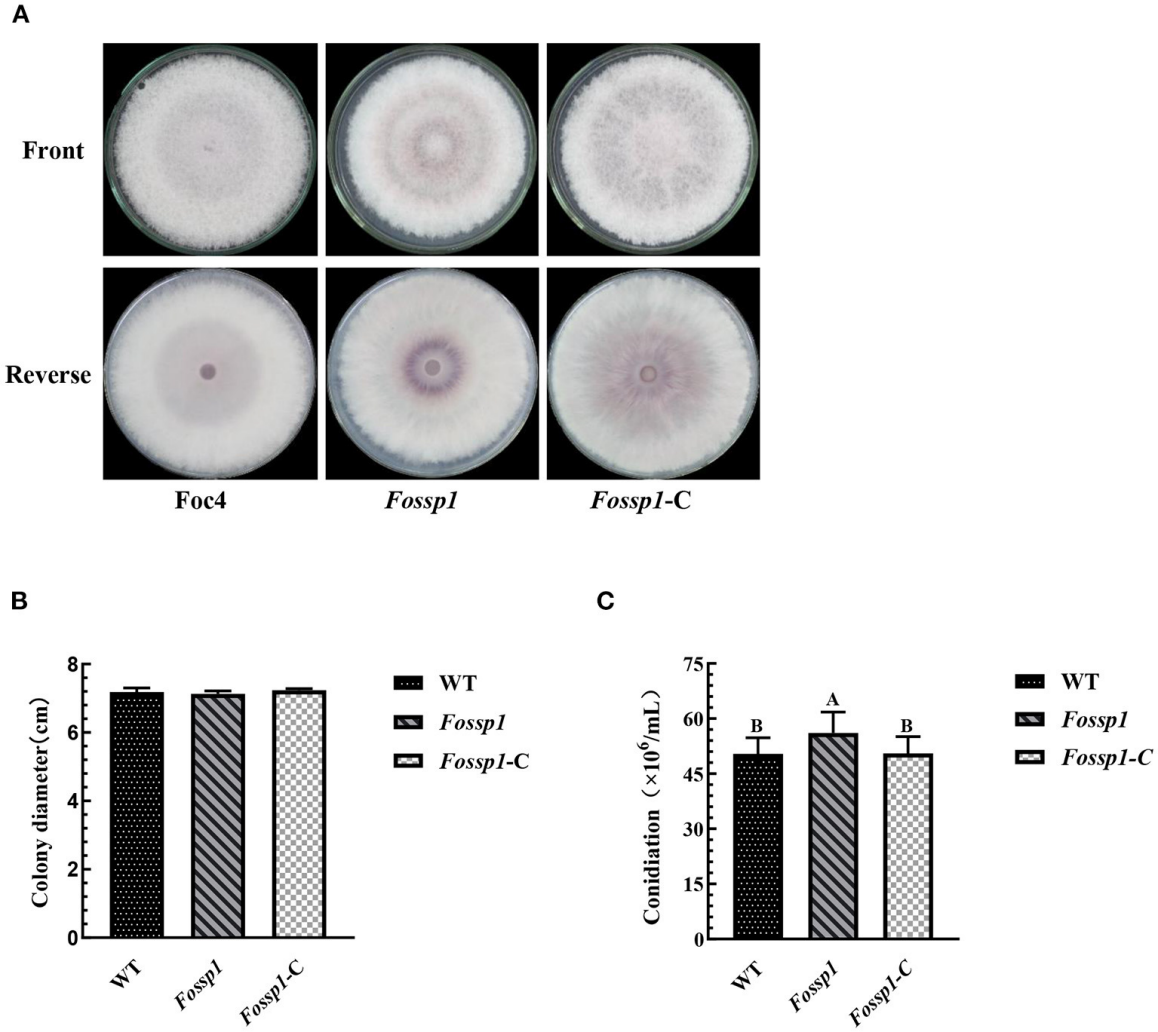
Effects of *Fossp1* deletion on colony morphology, growth rate, and spore yield of Foc4. **(A,B)** Colony morphology and growth rate of WT, *Fossp1*, and *Fossp1*-C cultures on PDA medium for 6 days. **(C)** Conidiation of WT, *Fossp1*, and *Fossp1*-C cultures in PDB medium for 2 days. The results are representative of three independent experiments. The error bars represent the standard error. Identical and conserved catalytic residues are marked with letters.

To examine the growth rate, wild-type strain of Foc4, *Fossp1* deletion mutant, and complement strain of *Fossp1*-C were cultured on PDA agar plates for 6 days. The results showed that no significant difference in growth rate among WT (0.59 ± 0.01 cm/d), *Fossp1*-C (0.61 ± 0.01 cm/d) and *Fossp1* deletion mutant (0.59 ± 0.01 cm/d) (Figures 4A,B).

The conidia production of Foc4, *Fossp1-*C, and *Fossp1* deletion mutant were calculated in PDB medium 2 days after inoculation. Interestingly, the conidial yield of *Fossp1* mutant (5.61 ± 0.35 × 10^5^ conidia/ml) was significantly higher than that of WT strain (5.04 ± 0.24 × 10^5^ conidia/ml) and complement strain *Fossp1-*C (5.05 ± 0.21 × 10^5^ conidia/ml) (Figure 4C). These results suggest that the *FoSSP1* gene regulates the sporulation of Foc4.

### Deletion Mutant of *Fossp1* Increased Virulence to Banana

**Figure 5:**
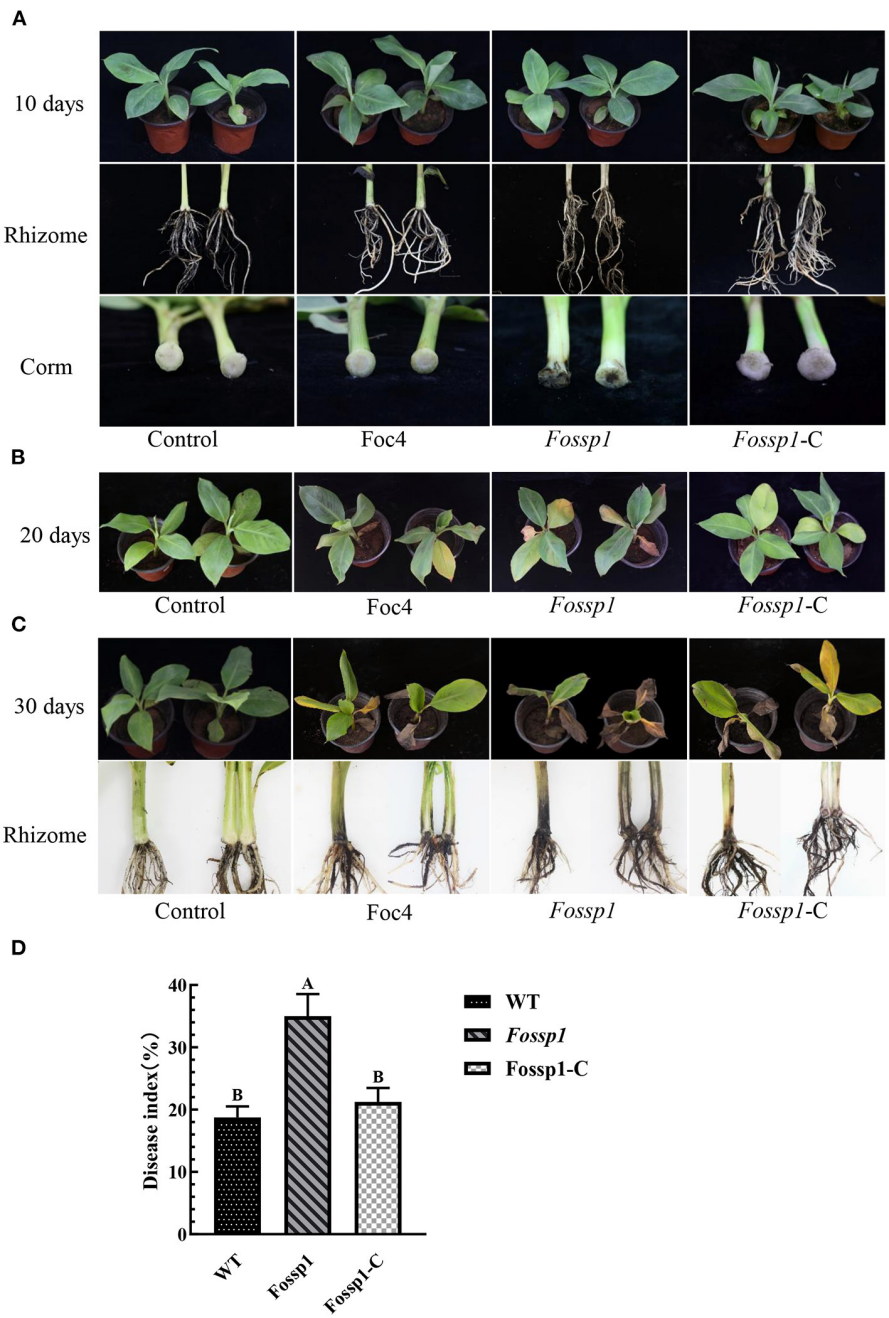
Deletion of *FoSSP1* increased the virulence of Foc4 on roots and rhizomes of bananas. Compared with the WT, the corms of banana seedlings inoculated with *Fossp1* showed obvious brown spots after 10 days **(A)**, and leaf yellowing appeared after 20 days **(B)**. The pseudostem and root displayed typical discoloration of infected seedlings after 30 days **(C)**. The experiment was repeated 3 times with 10 replicates for each of the three strains. **(D)** The disease index of bananas was scored 20 days post inoculation.

To analyze the pathogenicity of *FoSSP1*, it was knocked out and complementary successfully (Supplementary Figure S1). The knockout mutant *Fossp1*, complement strain *Fossp1-*C, and the WT Foc4 on banana seedlings (Cavendish banana var. Brazil (*Musa* AAA) was tested. The conidia suspension (1 × 10^8^ conidia/ml) of *Fossp1* mutant, complement strain *Fossp1-*C, and WT Foc4 were inoculated to the banana plantlets. At 10 dpi (days post inoculation), no symptoms presented on leaves and external bulbs of the *Fossp1, Fossp1*-C, and Foc4 (Figure 5A upper and middle panel). However typical discoloration internal of bulbs appeared when observed in the vertical section of the bulbs of *Fossp1* deletion mutant, but not of WT and complement strain (Figure 5A, lower panel). At 20 dpi, typical yellowing leaves of seedlings infected by Foc4 were observed. In comparing, two or more yellowing leaves of each seedling inoculated by the *Fossp1* deletion mutant, while of the wild-type and the complement strains yellowing leaves up to one of each seedling (Figure 5B). To quantify the disease severity of the infected banana seedlings with different Foc4 strains, the disease index of infected banana seedlings at 20 dpi was calculated. The disease index of banana plants infected with *Fossp1* deletion mutant was significantly increased than that of the WT strain Foc4 and complement *Fossp1*-C at 20 dpi (Figure 5D). When observed banana seedlings at 30 dpi, all plants infected by Foc4 displayed leaf yellowing, even the death of *Fossp1* (Figure 5C, upper panel). In addition, the pseudostem and root displayed typical discoloration of infected seedlings (Figure 5C, lower panel). Discoloration length of pseudostem was measured at 30 dpi, in comparison to WT Foc4 (4.15 ± 0.17 cm) and the complement strain (7.55 ± 0.31 cm), discoloration length of pseudostem of *Fossp1* (4.20 ± 0.30 cm) increased by about 50%. These results indicated that deletion of *FoSSP1* enhances the virulence of Foc4 in banana seedlings.

### *FoSSP1* Regulates the Expression of Foc4 Conidiation Regulator Genes and *Fossp1* Deletion Mutant Suppresses the Expression of Host Pathogenesis-Related Genes

**Figure 6:**
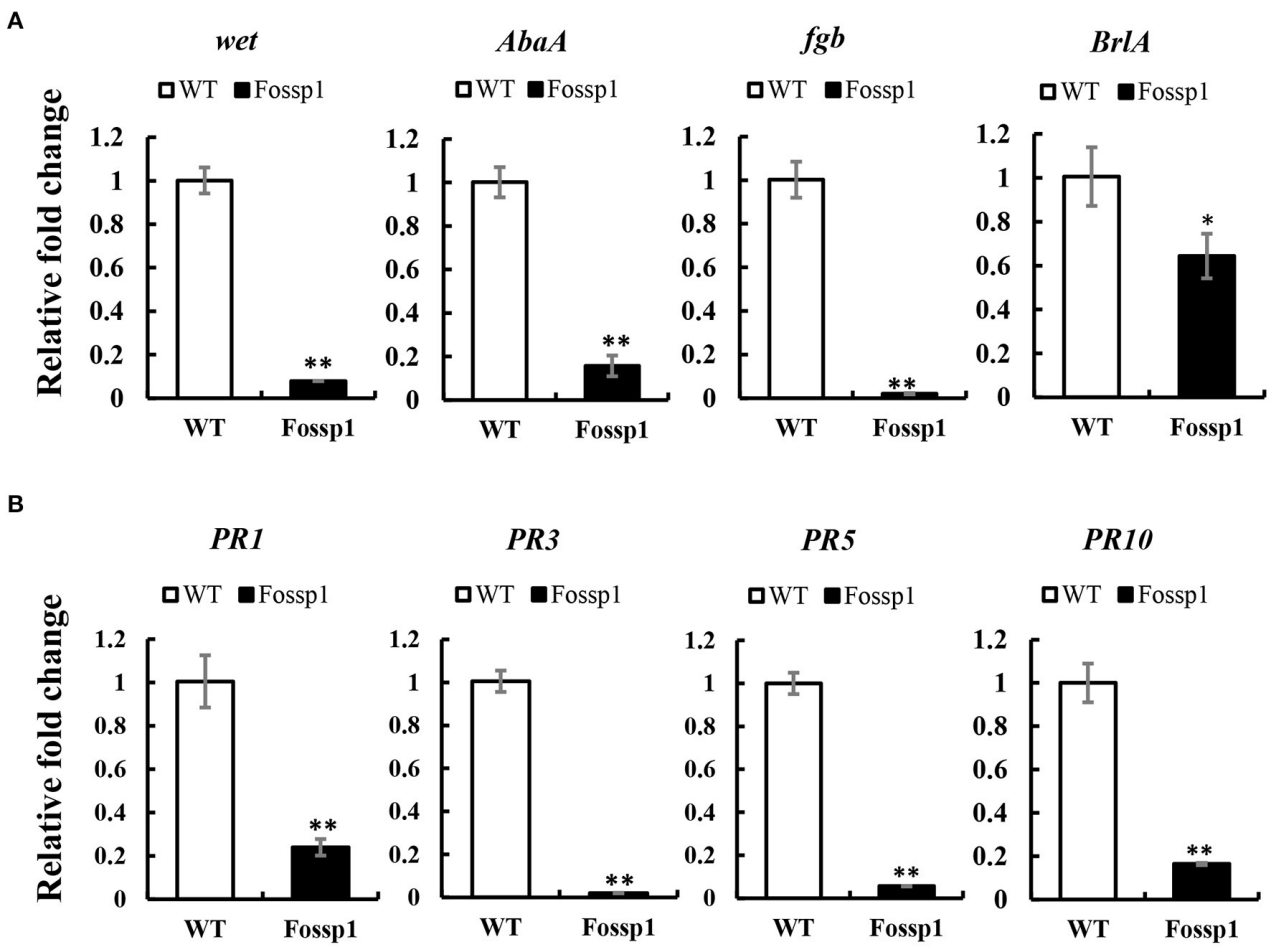
*FoSSP1* participates in the sporulation process and plant defense process. **(A)** The total RNAs were extracted from the spore suspension of WT and *Fossp1* cultured for 2 days, and the relative expression of four sporulation related genes was analyzed by quantitative reverse transcriptase-polymerase chain reaction (qRT-PCR), actin gene in Foc4 was used as reference gene. **(B)** The total RNAs were extracted from banana roots inoculated with WT and *Fossp1* spore suspension for 3 days, and the expression of 4 defense-related genes in banana was analyzed by qRT-PCR. Actin gene in banana was used as an internal reference gene in qRT-PCR. The experiment was repeated at least three times. The error bars represent the standard error. Identical and conserved catalytic residues are marked with asterisks (^**^).

Deletion of *FoSSP1* in Foc4 leads to the enhancement of sporulation capability. We hypothesized that *FoSSP1* might regulate Foc4 sporulation-related genes. Firstly, RNA of mycelia grown in a PDB medium for 2 days was extracted. Then, the transcription levels of the sporulation-related genes in WT and *Fossp1* deletion mutant were tested by quantitative reverse transcription PCR (qRT-PCR). Compared with the WT, the key genes in the sporulation pathway, *BrlA, Fgb, wet*, and *AbaA* were significantly down-regulated (Figure 6A).

Plant pathogenesis-related genes play a crucial role in plant defense against the pathogen (Joshi et al., 2021). Deletion of *Fossp1* to analyze differential expression of pathogenesis-related genes, the roots of banana seedlings were inoculated with a spore suspension of Foc4 and *Fossp1*, respectively, and total RNA was extracted 3 days post-inoculation to detect transcriptional levels of pathogenesis-related genes. RT-qPCR showed that the transcription level of PRs gene, *PR1b, PR2b, PR5*, and *PR10* in root infected by *Fossp1* was significantly lower than that did the WT (Figure 6B). The results of two qRT-PCR showed that *FoSSP1* induced the expression of host defense genes during the pathogen infection of roots. Furthermore, in the process of conidiation, *FoSSP1* participates in the central regulatory pathway controlling conidiation.

### The Predicted Secretory Peptide of FoSsp1 Is Functional

**Figure 7:**
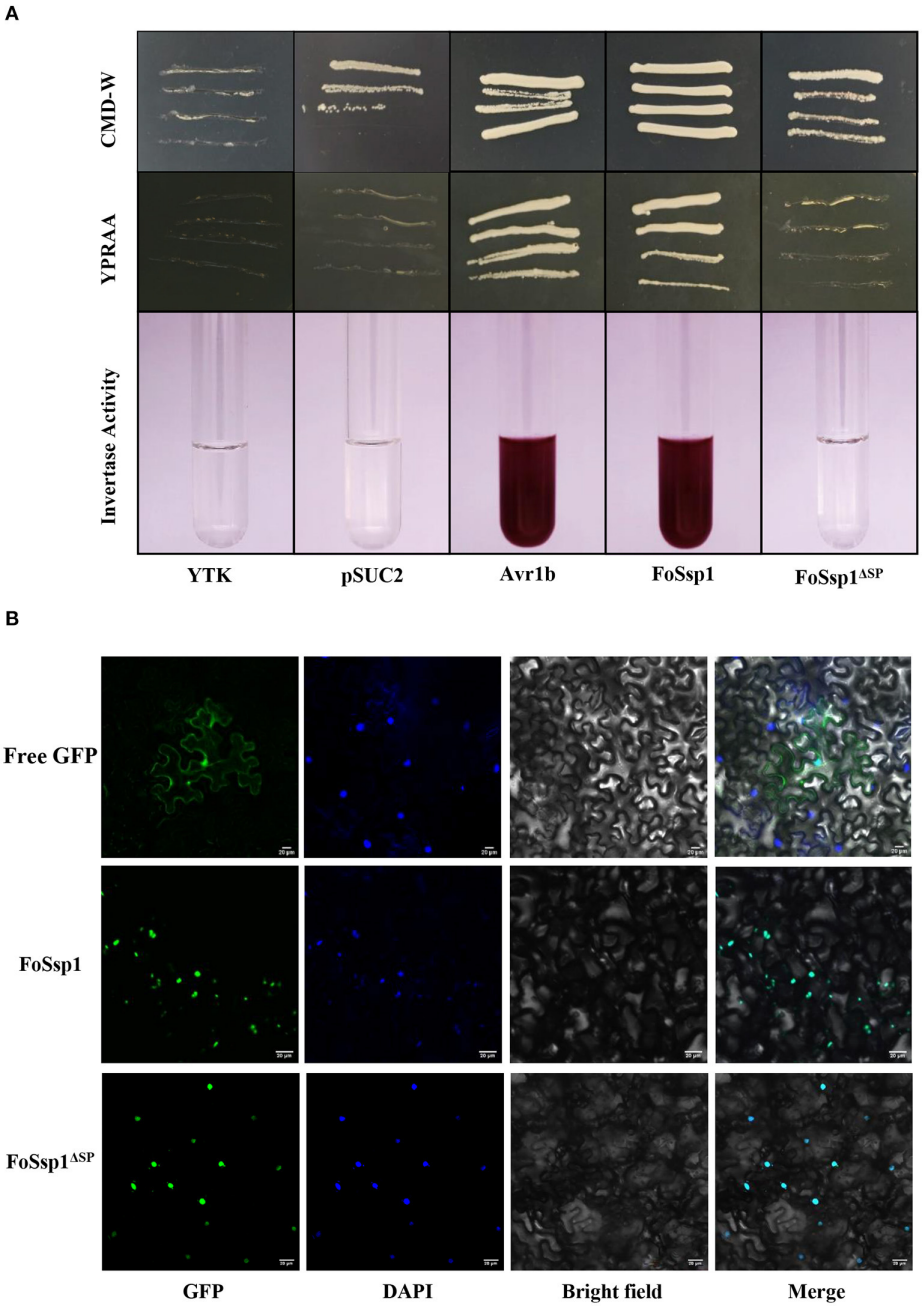
The functional validation of signaling peptide of FoSsp1 and the subcellular localization of FoSsp1 in tobacco epidermal cells. **(A)** Empty vector pSUC2 was used as negative control, SP sequence of Avr1b from *Phytophthora soja* was the positive control. The growth of yeast strains in the YPRAA and CMD-W medium were recorded. The change in color put down to the reduction of 2, 3, 5-triphenyltetrazolium chloride (TTC) to insoluble red-colored 1, 3, 5-triphenylformazan (TPF) was monitored. **(B)** DAPI used plant nuclear staining as nuclear control. The recombinant vector containing green fluorescent protein permeated tobacco leaves mediated by *A. tumefaciens*. Images were taken 2 days post infiltration. Bars, 20 μm. All experiments were repeated at least three times.

Secreted proteins can be divided into typical secreted proteins and atypical secreted proteins according to the presence or absence of signal peptides. A total of 23 amino acids (aa 1-23) at the N-terminal of FoSsp1 were predicted to be a signal peptide by SignalP5.0. The secretory function of the FoSsp1 signal peptide was verified using the yeast secretion system (Gu et al., 2011). The YTK12 strain was transformed with the reconstructed pSUC2 vector to express the signal peptide of pSUC2-FoSsp1 and pSUC2- FoSsp1^Δ*SP*^. The signal peptide of a known secreted protein, Avr1b, was used as a positive control (Tian et al., 2021). Untransformed YTK12 and empty pSUC2 vectors were used as negative controls. Similar to the Avr1b signal peptide, the FoSsp1 but not the FoSsp1^Δ*SP*^ or the empty vector, allowed YTK12 to grow on the YPRAA medium (Figure 7A). The strain transformed with the FoSsp1 with its signal peptide displayed secreted invertase enzyme activity that catalyzed the reduction of TTC to a red-colored compound (Figure 7A). These results suggest that FoSsp1 is a secreted protein, and its signal peptide is required for its secretion.

### Subcellular Localization of FoSsp1 in Tobacco Leaf Cell

To explore the subcellular localization of FoSsp1 in plant cells, the recombinant vector pBin-eGFP-*FoSSP1* and pBin-eGFP-*FoSSP*1^Δ*SP*^ were transiently expressed in tobacco leaves, and pBin-eGFP was used as a negative control. The plant nucleus was labeled with DAPI (2-(4-Amidinophenyl)-6-indolecarbamidine dihydrochloride) staining. When observed under the microscope, bright green fluorescence in the leaf cell nucleus of tobacco was overlapped with the blue fluorescence of the DAPI staining (Figure 7B). Interestingly, a weak fluorescence signal of pBin-eGFP-FoSSP1 was also distributed in extracellular and intracellular tobacco cells (Supplementary Figure S2). The result showed that FoSsp1 localized in the nucleus of a tobacco leaf cell.

### FoSsp1 Activates Plant Immune Response

**Figure 8:**
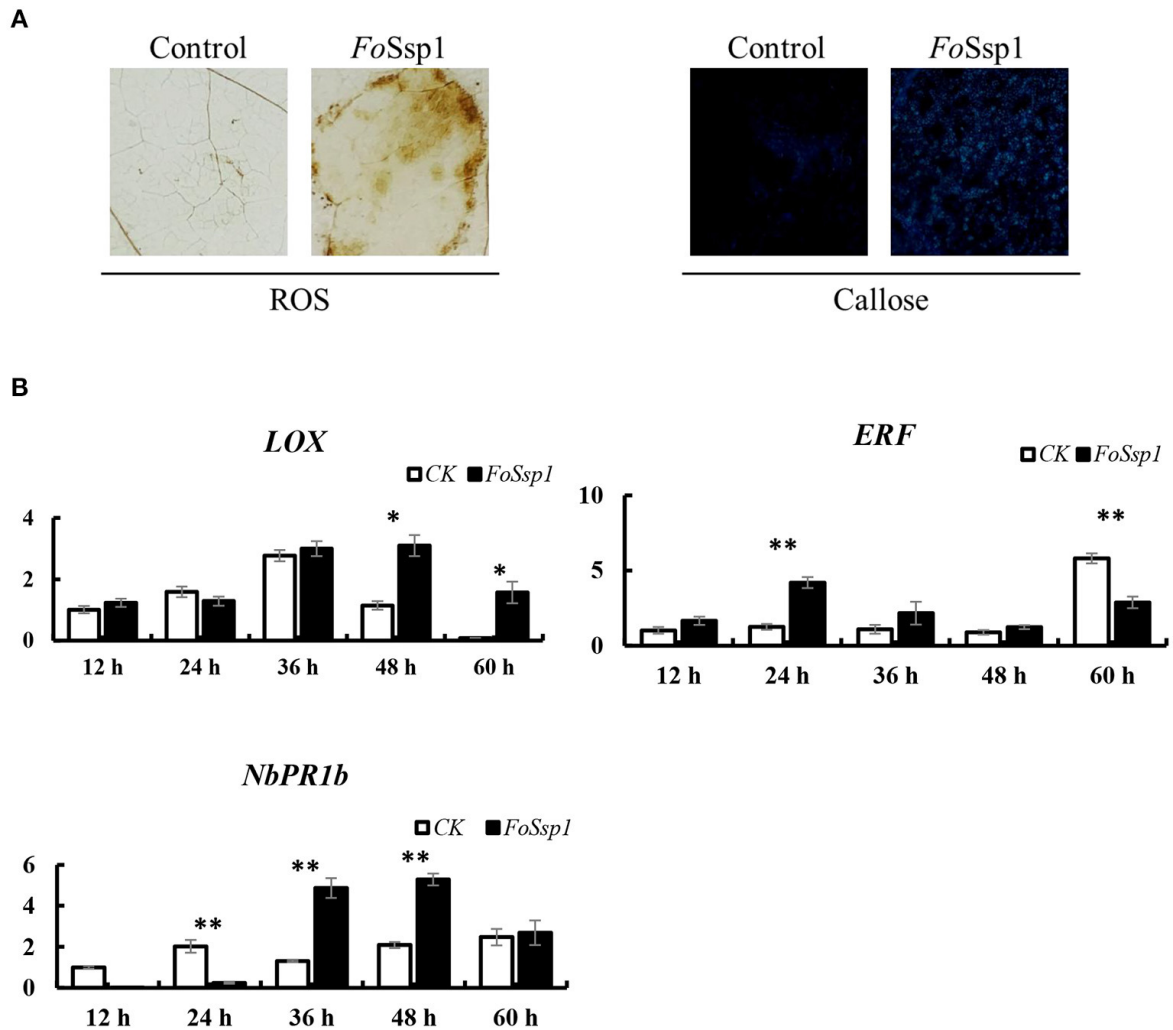
FoSsp1 can induce basic immune response in plants. **(A)** Tobacco leaves were infiltrated by *A. tumefaciens*-mediated transformation and stained with DAB and aniline blue 2 days post infiltration. Oxygen accumulation and callose depositions were visualized in tobacco leaves. The representative images were captured at 48 hpi. **(B)** The expression levels of 3 genes related to immune response were analyzed by qRT-PCR. The pBin-eGFP was the control group, and actin in tobacco was the internal reference gene. The expression of CK and FoSsp1 at the same time point were compared using one-way ANOVA. Asterisks represent significant differences in one-way ANOVA (*p* < 0.01). The results are representative of three independent experiments.

Due to the increased virulence phenotype of the *Fossp1* deletion mutant, we speculate that FoSsp1 is an elicitor that can stimulate the plant immune response. Therefore, the accumulation of H_2_O_2_ and the callose deposition in tobacco were detected after *A. tumefaciens* infiltration. FoSsp1 accumulates more H_2_O_2_ and callose than GFP control (Figure 8A). The results showed that FoSsp1 can activate the defense response of host plants, promoting oxygen burst and callose deposition.

To further explore the defense response of *N. benthamiana* activated by FoSsp1, the expression of defense-related genes was detected using qRT-PCR. The genes *pathogenesis-related protein 1B* (*PR1b*)*, pathogenesis-related protein 2B* (*PR2b*)*, ethylene-responsive transcription factor 1* (*ERF1*), and *linoleate 9S-lipoxygenase 5* (LOX) related to salicylic acid, ethylene, and jasmonic acid signal pathways were detected (Wang et al., 2000; Lorenzo et al., 2003; Wu et al., 2018). There were statistically significant differences in the expression of these genes. From the results, it is clear that PR1b and LOX showed higher expression levels at 48 hpi, while ERF1 expression significantly increased at 24 hpi compared with control (Figure 8B).

## Discussion

Fusarium wilt of bananas is a devastating soil-borne disease that has put the worldwide banana industry under threat (Dita et al., 2018). *F. oxysporum* f. sp. *cubense* race4 (Foc4) infects the root of banana, colonizes the vascular and blocks water and nutrient transportation, resulting in banana wilt (Chisholm et al., 2006). So far, however, genetic regulation of fungal virulence mechanisms remains elusive, with only a few virulence factors being reported. For example, loss of FGA1 and FGA3 resulted in decreased intracellular cyclic AMP levels and increased heat resistance, suggesting that FGA1 and FGA3 may regulate growth and development, pathogenicity, and heat resistance through the cAMP-dependent protein kinase A pathway (Guo et al., 2016). In this study, we identified a small secreted protein FoSsp1 that elicits plant defense reactions and negatively regulates plant infection. The results provide insights into fungal virulence mechanisms, and more importantly, offer implications for strategies aiming at sustainable control of fusarium wilt of bananas.

Small secretory proteins (SSPs) are important factors involved in plant host interaction (Xie et al., 2021). These proteins may facilitate infection by suppressing plant defenses, manipulating plant metabolism or signaling, or directly killing plant cells (Ramachandran et al., 2017). The identification and functional characterization of SIX8 support that SSPs could contribute toward the infection of vascular fungal pathogens as well (An et al., 2019). In this study, by combining bioinformatics analysis and functional analysis, we identified FoSsp1 to be a novel virulence-related SSP. Upon being transiently expressed in tobacco, FoSsp1 could induce cell death similar to BAX. Furthermore, FoSsp1 expression triggered obvious oxygen burst, callose accumulation, and the expressional up-regulation of plant immunity genes *PR1b, PR2b, Nb*LOX, and *Nb*ERF1. Corresponding with such defense-eliciting activities on tobacco, deletion of the *FoSSP1* gene increased fungal virulence toward bananas and reduced the expressional up-regulation of plant defense genes *PR1b, PR2b, PR5*, and *PR10*. Together, these results support that FoSsp1 is a novel defense-eliciting SSP that might enhance banana's resistance against fusarium wilt.

Elicitors can activate immune responses in plants and they can be engineered as plant immune inducers to preactivate the plant immune system to resist the invasion of pathogens, so as to promote plant disease resistance (Qiu et al., 2017; An et al., 2019). For example, BcGs1 enhanced bcGS1-induced resistance to *Botrytis cinerea* by activating basic plant defense responses and inducing lignin accumulation (Yang et al., 2018a). MoHrip2 stimulates the early defense response of rice seedlings through pressure-related pathways, induces up-regulated expression of the PR gene, activates SA and JA signaling pathways, and ultimately improves disease resistance of rice (Nie et al., 2019a). Identified elicitors are diverse in biochemical properties, and may be classified into oligosaccharides, lipids, aromatic compounds, or proteins (Shimizu et al., 2010; Ma et al., 2015; Clinckemaillie et al., 2017). A variety of elicitors are conserved molecular patterns that are indicative of pathogen presence and are perceived by plant cells via membrane-localized kinase receptors. For instance, PcCBP3 was labeled by mCherry and expressed in tobacco cells, and red fluorescence mainly was observed at the cell edge (Yin et al., 2021). Thus, elicitor perception represents an important plant immunity layer against pathogen infection. Functionally, FoSsp1 elicits plant defense reactions. However, our experimental evidence indicates that FoSsp1 might not function as a typical pathogen-associated molecular pattern (PAMP). First, deleting the predicted secretory peptide of FoSsp1 does not interfere with its death and defense triggering activity toward tobacco cells, indicating that the elicitor activity of FoSsp1 relies on perturbing intracellular plant cell activity. During infection, FoSsp1 might translocate inside a plant cell to exert elicitor functions. In accordance with this, our GFP-based subcellular localization experiment demonstrates FoSsp1 localizes within the nucleus upon transiently expressed on tobacco. Besides its subcellular localization, FoSsp1 also differs from a typical PAMP in that its deletion does not negatively affect pathogen growth, development, and virulence, indicating that *FoSSP1* might be dispensable for the fungal pathogen. As said, it is worth noting that *FoSSP1* homologs are widespread in ascomycetes, indicating a conserved biological function. Indeed, maintaining a defense-eliciting elicitor would be evolutionarily disadvantageous for a plant pathogen unless the elicitor provides additional benefits for the pathogen. In this study, deletion of *FoSSP1* caused elevated conidia production, indicating that *FoSSP1* may regulate proper conidial differentiation. In the future, characterizing the functions of *FoSSP1* homologs in other fungi would be important to unravel the potentially multifaceted biological functions of the SSP family.

The identification of an elicitor protein from the banana-*Fusarium* pathosystem offers important insights for the sustainable control of fusarium wilt disease. Due to the cloning propagation nature of bananas, the natural banana population is the shortage of genetic diversity and the breeding utilization of potential natural resistance is technically challenging (Nyine et al., 2017), which has added considerable difficulties to the sustainable control of banana fusarium wilt disease. In the future, it might be feasible to take advantage of the elicitor activity of FoSsp1 for developing transgene plants or developing bioproducts with activated elicitor.

## Data Availability Statement

The datasets presented in this study can be found in online repositories. The names of the repository/repositories and accession number(s) can be found in the article/Supplementary Material.

## Author Contributions

DC and LZ designed the experiments. YW, XZ, and TW performed the experiments. YW, SZ, XL, CX, and ZK analyzed the data. XL, DC, and LZ joined the discussion and gave the original ideas. YW, DC, and LZ wrote the paper. All authors contributed to the article and approved the submitted version.

## Funding

This study was financially supported by Hainan Provincial Natural Science Foundation of China (no. 320QN188), the Finance Science and Technology Project of Hainan Province (no. YSPTZX202018), Hainan Major Research Fund of Science and Technology (no. ZDKJ201817), Funding for the Construction of World First Class Discipline of Hainan University (no. RZZX201911), and the Scientific Research Foundation for Advanced Talents, Hainan University (no. KYQD(ZR)1873).

## Conflict of Interest

The authors declare that the research was conducted in the absence of any commercial or financial relationships that could be construed as a potential conflict of interest.

## Publisher's Note

All claims expressed in this article are solely those of the authors and do not necessarily represent those of their affiliated organizations, or those of the publisher, the editors and the reviewers. Any product that may be evaluated in this article, or claim that may be made by its manufacturer, is not guaranteed or endorsed by the publisher.
