# Supplementary material for: The Small Secreted Protein FoSsp1 Elicits Plant Defenses and Negatively Regulates Pathogenesis in Fusarium oxysporum f. sp. cubense (Foc4)
Source: Front Plant Sci. 2022 May 10;13:873451. doi: 10.3389/fpls.2022.873451 (PMC9129915; doi:10.3389/fpls.2022.873451)
Supplement: Supplementary file 1 [file Data_Sheet_1.docx]

Supplementary Material

The following supplementary materials are available for this article:


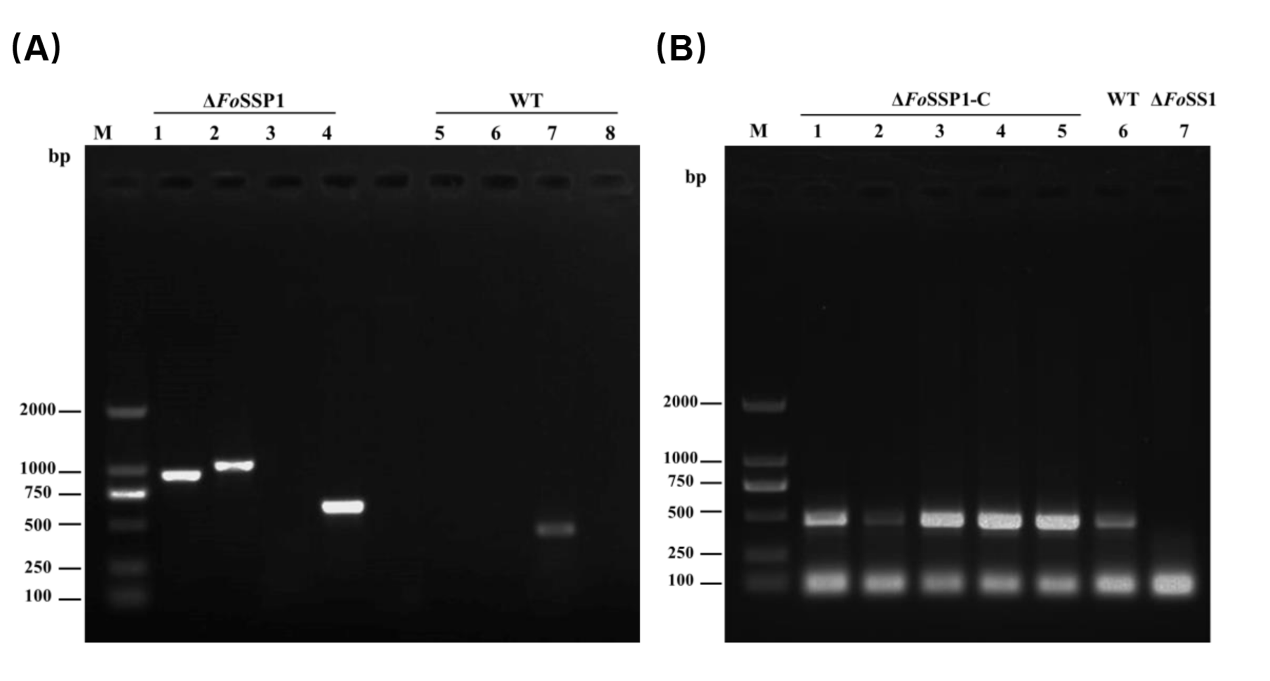


Figure S1. PCR Test of *Fossp1* deletion mutants. (A) Part of *FoSSP1* and hygromycin resistance gene sequences were amplified with NF/NR and H850R/H852F (Table S1), respectively (lane 3, 7; 4, 8). The upstream of *FoSSP1* overlapped trpC promoter was amplified with primer PF/H850R (Lanes 1, 5). The trpC terminator overlapped downstream of *FoSSP1* was amplified with primers H852F/PR (Lanes 2, 6). (B) The full-length sequence of *FoSSP1* was obtained by SSP1-S-F/R. Lane M, DL2000 marker.


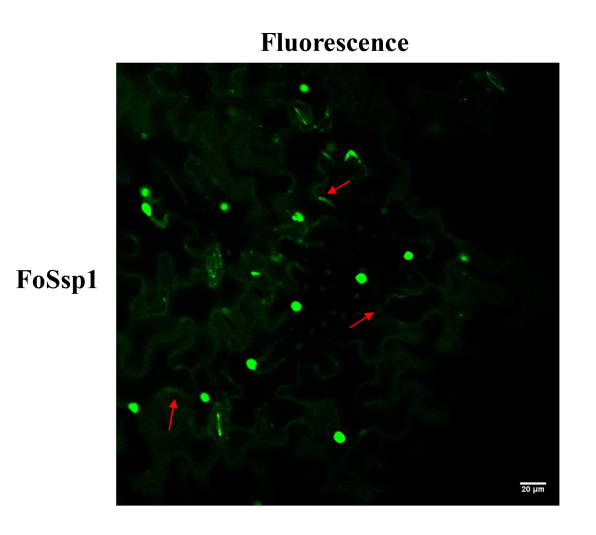


Figure S2. Subcellular localization of FoSsp1 in tobacco epidermal cells. The recombinant vector containing full-length of FoSsp1 permeated tobacco leaves mediated by *A. tumefaciens*. The arrow points to the weak green fluorescence at the edge of the cell. Images were taken 2 days post infiltration. Bars, 20 µm.

**Table S1. Polynucleotide chain reaction primers used in this study.**

| Primer | Sequence（5’-3’） |
| --- | --- |
| UF | GGGTTCCCCCAAGACTGAT |
| UR | TTGACCTCCACTAGCTCCAGCCAAGCCGCCCCGTAATGCGTTTCAA |
| DF | GAATAGAGTAGATGCCGACCGCGGGTTCCCACGAAGAAATGAACGCC |
| DR | CTTCAGGCAACAAGCACGG |
| NF | GCAATCAGCCCCTCCTTT |
| NR | CTCATTGCCGCTTCCATC |
| PF | ATGGCTTGGGCAAATGAG |
| PR | ACAACCCATCAGCAGGCA |
| YG-F | GATGTAGGAGGGCGTGGATATGTCCT |
| HY-R | GTATTGACCGATTCCTTGCGGTCCGAA |
| HYG-F | GGCTTGGCTGGAGCTAGTGGAGGTCAA |
| HYG-R | AACCCGCGGTCGGCATCTACTCTATTC |
| H850R | TTGTCCGTCAGGACATTGTT |
| H852F | AACTCACCGCGACGTCTGTC |
| SSP1-S-F | CGGAATTTTAATTAAGAATTCATGTTGTTTCAGAATATCCTCATTGC |
| SSP1-S-R | CACTATAGGGAGAACCTCGAGCTACAGGCAAATTCCAGAGCAAT |
| Actin-RT-F | GAGGGCGAGGTATTCAT |
| Actin-RT-R | TGGATGTGTGGAGTGCG |
| BActin-F | ACCGAGGCTCCCATCAACC |
| BActin-R | CGGTGGTGGAGAAAGTGTAA |
| NActin-F | TGTGAAGGAGAAGTTGGCT |
| NActin-R | GAATCTCTCAGCACCAATG |
| SSP1-RT-F | CTCATTGCCGCTTCCAT |
| SSP1-RT-R | CGAGGGTCATCAACACAGT |
| PR1-F | GCCTTACGGGGAAAACTT |
| PR1-R | CAGGCTAAGTTTTCCCCG |
| PR3-F | ACGCCTTTATCACCGCTG |
| PR3-R | TAGCGTTTGCCAGATGCG |
| PR5-F | CAAGCGGCATTGCTGTTA |
| PR5-F | CAAGCGGCATTGCTGTTA |
| PR10-F | CAGCGTCAGGCAGTTCAA |
| PR10-R | TCAGTTGTAGGCGTCCG |
| BrlA-F | GACTCTCTCCCCAACACTC |
| BrlA-R | AGATGGGACTATCGTTGGC |
| Aba-F | AACAACAGCCGTCTACCTC |
| AbaA-R | AGGGTTGACACCATTTCTG |
| wet-F | CAATCCCCTCTCAGCCA |
| wet-R | GGTTGGCGATGATGGTT |
| fgb-F | GCCAAGCGAACACTGAA |
| fgb-R | CGCAAGCCACGAAGTTA |
| PR1b-F | TTGGATGCCCATAACACAG |
| PR1b-R | ATAAAATCGCCACTTCCCT |
| PR2b-F | AACTAAGAATGCGGAAAGC |
| PR2b-R | TCTGGGGAGTAGATTCGGA |
| ERF1-F | GCGGTTCAAAGGCTCA |
| ERF1-R | ACAGCCTTTCTTCTCCGTT |
| LOX-F | CTCGTAGAGGCAGGACA |
| LOX-R | TCGTCCCTTGGCACATA |

**Table S2. Small secreted proteins screened in this study.**

| Protein ID | SP(Sec/SPI) | TMHs | GPI-modification site | | Length | Cysteine number | Cysteine level |
| --- | --- | --- | --- | --- | --- | --- | --- |
| XP_031052215.1 | 99.78% | None | None | 118 | | 7 | 5.93% |
| XP_031052273.1 | 99.89% | None | None | 140 | | 8 | 5.71% |
| XP_031052559.1 | 99.93% | None | None | 357 | | 8 | 2.24% |
| XP_031052717.1 | 99.08% | None | None | 230 | | 4 | 1.74% |
| XP_031053310.1 | 99.80% | None | None | 374 | | 7 | 1.87% |
| XP_031053315.1 | 99.40% | None | None | 488 | | 8 | 1.64% |
| XP_031053463.1 | 99.88% | None | None | 127 | | 8 | 6.30% |
| XP_031053644.1 | 93.80% | None | None | 598 | | 8 | 1.34% |
| XP_031053645.1 | 93.80% | None | None | 461 | | 7 | 1.52% |
| XP_031053664.1 | 99.90% | None | None | 98 | | 8 | 8.16% |
| XP_031054034.1 | 98.19% | None | None | 306 | | 4 | 1.31% |
| XP_031054110.1 | 99.13% | None | None | 234 | | 4 | 1.71% |
| XP_031054128.1 | 99.39% | None | None | 553 | | 12 | 2.17% |
| XP_031054214.1 | 99.83% | None | None | 377 | | 6 | 1.59% |
| XP_031054274.1 | 99.94% | None | None | 145 | | 4 | 2.76% |
| XP_031054384.1 | 99.72% | None | None | 679 | | 7 | 1.03% |
| XP_031054563.1 | 88.04% | None | None | 371 | | 8 | 2.16% |
| XP_031054686.1 | 99.84% | None | None | 267 | | 11 | 4.12% |
| XP_031054957.1 | 96.26% | None | None | 297 | | 7 | 2.36% |
| XP_031054967.1 | 99.98% | None | None | 382 | | 6 | 1.57% |
| XP_031054997.1 | 94.66% | None | None | 499 | | 8 | 1.60% |
| XP_031055016.1 | 98.48% | None | None | 298 | | 10 | 3.36% |
| XP_031055022.1 | 99.77% | None | None | 460 | | 7 | 1.52% |
| XP_031055044.1 | 52.12% | None | None | 599 | | 6 | 1.00% |
| XP_031055170.1 | 99.20% | None | None | 298 | | 13 | 4.36% |
| XP_031055337.1 | 98.34% | None | None | 384 | | 7 | 1.82% |
| XP_031055425.1 | 96.66% | None | None | 344 | | 4 | 1.16% |
| XP_031055446.1 | 94.86% | None | None | 231 | | 17 | 7.36% |
| XP_031055551.1 | 98.80% | None | None | 377 | | 7 | 1.86% |
| XP_031055585.1 | 98.15% | None | None | 124 | | 4 | 3.23% |
| XP_031055641.1 | 99.00% | None | None | 140 | | 8 | 5.71% |
| XP_031056043.1 | 99.68% | None | None | 227 | | 6 | 2.64% |
| XP_031056440.1 | 99.26% | None | None | 571 | | 7 | 1.23% |
| XP_031057122.1 | 55.49% | None | None | 455 | | 10 | 2.20% |
| XP_031057988.1 | 96.80% | None | None | 550 | | 13 | 2.36% |
| XP_031057997.1 | 99.24% | None | None | 89 | | 4 | 4.49% |
| XP_031058044.1 | 99.89% | None | None | 75 | | 4 | 5.33% |
| XP_031058096.1 | 99.91% | None | None | 384 | | 7 | 1.82% |
| XP_031058103.1 | 99.25% | None | None | 566 | | 8 | 1.41% |
| XP_031058280.1 | 78.86% | None | None | 582 | | 8 | 1.37% |
| XP_031058289.1 | 99.80% | None | None | 298 | | 14 | 4.70% |
| XP_031058407.1 | 99.68% | None | None | 138 | | 8 | 5.80% |
| XP_031058421.1 | 99.81% | None | None | 650 | | 9 | 1.38% |
| XP_031058690.1 | 99.60% | None | None | 126 | | 6 | 4.76% |
| XP_031059215.1 | 95.64% | None | None | 595 | | 7 | 1.18% |
| XP_031059273.1 | 99.42% | None | None | 247 | | 6 | 2.43% |
| XP_031059371.1 | 97.83% | None | None | 137 | | 8 | 5.84% |
| XP_031059402.1 | 99.02% | None | None | 248 | | 6 | 2.42% |
| XP_031059425.1 | 99.56% | None | None | 131 | | 4 | 3.05% |
| XP_031059465.1 | 96.85% | None | None | 334 | | 4 | 1.20% |
| XP_031059473.1 | 99.47% | None | None | 447 | | 11 | 2.46% |
| XP_031059475.1 | 99.30% | None | None | 231 | | 4 | 1.73% |
| XP_031059479.1 | 83.12% | None | None | 664 | | 9 | 1.36% |
| XP_031059579.1 | 94.58% | None | None | 233 | | 13 | 5.58% |
| XP_031059644.1 | 99.89% | None | None | 276 | | 7 | 2.54% |
| XP_031059646.1 | 98.84% | None | None | 361 | | 9 | 2.49% |
| XP_031059654.1 | 98.75% | None | None | 312 | | 4 | 1.28% |
| XP_031059664.1 | 96.52% | None | None | 356 | | 4 | 1.12% |
| XP_031059802.1 | 98.85% | None | None | 536 | | 11 | 2.05% |
| XP_031059947.1 | 99.05% | None | None | 558 | | 15 | 2.69% |
| XP_031060184.1 | 69.34% | None | None | 297 | | 6 | 2.02% |
| XP_031060198.1 | 95.84% | None | None | 371 | | 10 | 2.70% |
| XP_031060201.1 | 99.54% | None | None | 257 | | 4 | 1.56% |
| XP_031060240.1 | 98.73% | None | None | 109 | | 4 | 3.67% |
| XP_031060303.1 | 88.33% | None | None | 275 | | 7 | 2.55% |
| XP_031060352.1 | 71.76% | None | None | 658 | | 9 | 1.37% |
| XP_031060638.1 | 78.75% | None | None | 549 | | 11 | 2.00% |
| XP_031060859.1 | 93.35% | None | None | 227 | | 10 | 4.41% |
| XP_031060911.1 | 99.65% | None | None | 132 | | 8 | 6.06% |
| XP_031061288.1 | 99.46% | None | None | 254 | | 10 | 3.94% |
| XP_031061615.1 | 99.35% | None | None | 378 | | 8 | 2.12% |
| XP_031061626.1 | 98.65% | None | None | 410 | | 7 | 1.71% |
| XP_031061815.1 | 77.96% | None | None | 335 | | 4 | 1.19% |
| XP_031061816.1 | 99.38% | None | None | 406 | | 13 | 3.20% |
| XP_031061942.1 | 93.81% | None | None | 416 | | 5 | 1.20% |
| XP_031062265.1 | 90.52% | None | None | 394 | | 4 | 1.02% |
| XP_031062547.1 | 98.13% | None | None | 360 | | 9 | 2.50% |
| XP_031062632.1 | 94.33% | None | None | 321 | | 7 | 2.18% |
| XP_031062649.1 | 99.72% | None | None | 313 | | 5 | 1.60% |
| XP_031062753.1 | 95.92% | None | None | 200 | | 4 | 2.00% |
| XP_031063603.1 | 89.38% | None | None | 378 | | 16 | 4.23% |
| XP_031064095.1 | 99.66% | None | None | 139 | | 4 | 2.88% |
| XP_031064096.1 | 99.66% | None | None | 139 | | 4 | 2.88% |
| XP_031064189.1* | 99.13% | None | None | 145 | | 18 | 12.41% |
| XP_031064269.1 | 83.14% | None | None | 409 | | 8 | 1.96% |
| XP_031064270.1 | 83.14% | None | None | 409 | | 8 | 1.96% |
| XP_031064741.1 | 87.99% | None | None | 362 | | 6 | 1.66% |
| XP_031065561.1 | 97.15% | None | None | 378 | | 4 | 1.06% |
| XP_031066528.1 | 99.53% | None | None | 240 | | 8 | 3.33% |
| XP_031067043.1 | 56.71% | None | None | 435 | | 6 | 1.38% |
| XP_031068235.1 | 98.47% | None | None | 328 | | 6 | 1.83% |
| XP_031068963.1 | 98.95% | None | None | 276 | | 5 | 1.81% |
| XP_031069417.1 | 99.90% | None | None | 249 | | 3 | 1.20% |
| XP_031070534.1 | 99.67% | None | None | 651 | | 10 | 1.54% |
| XP_031070624.1 | 96.12% | None | None | 406 | | 5 | 1.23% |
| XP_031070698.1 | 99.79% | None | None | 100 | | 8 | 8.00% |
| XP_031070905.1 | 99.87% | None | None | 144 | | 4 | 2.78% |
| XP_031070912.1 | 99.96% | None | None | 429 | | 18 | 4.20% |
| XP_031070930.1 | 96.05% | None | None | 235 | | 4 | 1.70% |
| XP_031070931.1 | 91.00% | None | None | 430 | | 5 | 1.16% |
| XP_031070970.1 | 99.23% | None | None | 350 | | 8 | 2.29% |
| XP_031071946.1 | 95.35% | None | None | 375 | | 7 | 1.87% |
| XP_031073283.1 | 99.47% | None | None | 514 | | 24 | 4.67% |

*Eleven proteins selected for transient expression in tobacco leaves was highlitghed with yellow.
